# Periostin levels and eosinophilic inflammation in poorly-controlled asthma

**DOI:** 10.1186/s12890-016-0230-4

**Published:** 2016-04-30

**Authors:** Jodie L. Simpson, Ian A. Yang, John W. Upham, Paul N. Reynolds, Sandra Hodge, Alan L. James, Christine Jenkins, Matthew J. Peters, Guiquan Jia, Cecile T. J. Holweg, Peter G. Gibson

**Affiliations:** Department of Respiratory and Sleep Medicine, Hunter Medical Research Institute, Level 2, West Wing, Lot 1 Kookaburra Circuit, New Lambton Heights, NSW 2305 Australia; Priority Research Centre for Asthma and Respiratory Disease, The University of Newcastle, Newcastle, NSW Australia; School of Medicine, The University of Queensland, Brisbane, QLD Australia; Department of Thoracic Medicine, The Prince Charles Hospital, Brisbane, QLD Australia; Department of Respiratory Medicine, Princess Alexandra Hospital, Brisbane, QLD Australia; Department of Thoracic Medicine, Royal Adelaide Hospital, Adelaide, SA Australia; Lung Research Laboratory, Hanson Institute, Adelaide, SA Australia; Department of Pulmonary Physiology and Sleep Medicine, Sir Charles Gairdner Hospital, Nedlands, WA Australia; School of Medicine and Pharmacology, The University of Western Australia, Crawley, WA Australia; Respiratory Trials, The George Institute for Global Health, Sydney, NSW Australia; Australian School of Advanced Medicine, Macquarie University, Sydney, NSW Australia; Department of Thoracic Medicine, Concord General Hospital, Concord, NSW Australia; Genentech Inc, South San Francisco, CA USA; Woolcock Institute of Medical Research, Glebe, NSW Australia

**Keywords:** Periostin, Eosinophilic asthma, Non-eosinophillic asthma, Phenotype, Inflammation

## Abstract

**Background:**

Periostin levels are associated with airway eosinophilia and are suppressed by corticosteroid treatment in asthma. This study sought to determine the relationship between serum and sputum periostin, airway inflammatory phenotype and asthma control.

**Methods:**

Adults with poorly-controlled asthma (*n* = 83) underwent a clinical assessment, sputum induction and blood sampling. Dispersed sputum was used for a differential cell count and periostin assessment (ELISA). Serum periostin was determined by the Elecsys® immunoassay.

**Results:**

Periostin levels were significantly higher in serum (median (IQR) of 51.6 (41.8, 62.6) ng/mL) than in sputum (1.1 (0.5, 2.0) ng/mL) (*p* < 0.001). Serum and sputum periostin were significantly higher in patients with eosinophilic asthma (*n* = 37) compared with non-eosinophilic asthma. Both serum and sputum periostin levels were significantly associated with proportion of sputum eosinophils (*r* = 0.422, *p* < 0.001 and *r* = 0.364, *p* = 0.005 respectively) but were not associated with asthma control. In receiver operator characteristic curve analysis, the area under the curve (AUC) for serum periostin (*n* = 83) was 0.679, *p* = 0.007. Peripheral blood eosinophils assessed in 67 matched samples, had a numerically greater AUC of 0.820 compared with serum periostin, *p* = 0.086 for the detection of eosinophilic asthma.

**Conclusion:**

In poorly-controlled asthma, sputum and serum periostin levels are significantly related to sputum eosinophil proportions while their ability to predict the presence of eosinophilic asthma is modest.

**Electronic supplementary material:**

The online version of this article (doi:10.1186/s12890-016-0230-4) contains supplementary material, which is available to authorized users.

## Background

Asthma is a chronic heterogeneous inflammatory disorder of the airways where approximately 50 % of adults have eosinophilic airway inflammation which is referred to as eosinophilic asthma. Sputum eosinophils are an effective monitoring tool in asthma when used in therapeutic decision making and there is good evidence that this approach can significantly reduce severe exacerbations [1, 2]. The technical difficulties associated with sputum collection and processing limit the use of this management strategy in clinical practice. Alternative and accurate biomarkers that can predict sputum eosinophils are needed to facilitate inflammation-based asthma management.

We have recently shown that blood eosinophil counts are a suitable surrogate for sputum eosinophil proportion in identifying patients with eosinophilic asthma, being simple and readily available and thus may provide a suitable asthma management tool [3]. Similarly, reports also show that serum periostin levels are associated with airway eosinophilia in asthma [4]. In bronchial brushings, periostin gene expression is upregulated in a subset of patients in which it was associated with the expression of Th2 cytokines. These patients also had higher blood and BAL eosinophil counts and were most responsive to inhaled corticosteroids (ICS) [5]. Periostin is expressed by lung epithelial cells and fibroblasts and in-vitro experiments show that periostin is secreted towards the basolateral side of the airway [6, 7]. However little is known about the airway levels of periostin and the relationships between airway periostin levels, asthma inflammatory subtypes and asthma control.

This study sought to examine the ability of serum periostin to predict the presence of eosinophilic asthma and to determine the relationship between airway and serum periostin levels, inflammatory subtype and asthma control in a group of adults with poorly-controlled asthma.

## Methods

### Study participants

Eligible adults with asthma (*n* = 83) who were participants in a randomised controlled trial of macrolides in asthma. The diagnosis of asthma was established using the American Thoracic Society guidelines based upon the presence of current episodic respiratory symptoms (past 12 months), doctor-confirmed diagnosis and evidence of variable airflow obstruction [8] (airway hyperresponsiveness, bronchodilator response or diurnal variation of peak expiratory flow). They also were prescribed maintenance ICS treatment and remained poorly-controlled with an Asthma Control Questionnaire 6 (ACQ6) score >0.7 [9]. Participants with an FEV_1_ < 40 % predicted, current smokers, ex-smokers who had ceased smoking in the previous year and those with an exacerbation or respiratory infection in the past four weeks were excluded. Those with significant smoking-related emphysema (ex-smokers >10 pack year history and carbon monoxide transfer coefficient <70 % predicted) OR smoking history >10 pack years and exhaled carbon monoxide >10 ppm were also excluded.

Participants underwent a clinical assessment, allergy skin test, spirometry with bronchodilator response, sputum induction and blood sampling in turn. All tests were performed on the same day prior to the commencement of study medication. Measurements were carried out by observers blinded to other results.

### Ethics, consent and permissions

Subjects gave written informed consent (Approval to the protocol was provided by the following ethics committee’s: Sir Charles Gairdner Group Human Research ethics committee (2008-147), Royal Adelaide Hospital Human Research Ethics Committee (081108f), Hunter New England Research Ethics Committee (08/11/19/3.03), The Prince Charles Hospital Metro North Hospital and Health Services (HREC/08/QPCH/4), Metro South Health Service District Human Research Ethics Committee (HREC/09/QPAH/015).

### Study design

This cross-sectional study characterised participants during a single visit where symptoms, atopy, medication use and smoking status were assessed, and the spirometry and sputum induction and blood collection were undertaken. Sputum was processed for inflammatory cell counts and sputum supernatant and serum were stored for periostin analysis.

### Sputum induction and analysis

Spirometry (CPFS/D™ USB Spirometer, BreezeSuite™ v7.1, MGC Diagnostics, Saint Paul, MN, USA) and induced sputum were performed. Sputum was induced by the inhalation of hypertonic saline (4.5 %) as described by Gibson et al. [10]. Subjects received a mean nebulization time of 13.7 min.

Sputum was processed as described [10]. Selected sputum portions were dispersed by dithiothreitol (DTT) (Merck Millipore, Darmstadt, Germany) and a total cell count performed. Cytospins were prepared and stained with May-Grünwald Giemsa. Cell counts were performed on 400 non-squamous cells. Cell viability and differential cell counts were recorded.

Asthma subtypes were defined by percentages of sputum eosinophils and neutrophils (of total inflammatory cells) and were classified as eosinophilic asthma if sputum eosinophils were ≥3 % and sputum neutrophils were <61 %, as previously defined [11].

### Blood samples and periostin serum assessment

Venous blood samples were collected without anticoagulant. Periostin levels in serum were measured using the clinical trial version of the Elecsys® Periostin immunoassay (Roche Diagnostics, Penzberg, Germany) intended for use on the cobas e 601 immunology analyzer, an automated electrochemiluminescence immunoassay based on the sandwich principle [12]. Peripheral blood eosinophil counts were assessed in 67 participants which are part of a previously published dataset [3].

### Periostin sputum assessment

Periostin levels in sputum were measured using the Genentech proprietary ELISA using the same antibody pair as the Elecsys® assay [4]. The limit of detection of the assay is 9.4 pg/mL.

### Analysis

The results were expressed as mean ± SD for continuous variables and median with interquartile range (IQR) when data were not normally distributed. Categorical data were reported using frequencies and percentages. A Kruskal-Wallis test was performed in the different subgroups of subjects with asthma. Spearman’s rank correlation coefficient was used to assess the associations between non-parametric data. Results were reported as significant when *p* < 0.05. The performance characteristics of the periostin variables were examined by receiver operating characteristic (ROC) curves to determine the concentrations which best defined eosinophilic asthma based on the sputum cell count. Participants were assigned to the high periostin group if their serum periostin levels were ≥50 ng/mL [13].

## Results

Participants consisted of 83 adults with a mean age of 61 years who were predominantly atopic (73 %) and poorly-controlled (mean ACQ6 1.83) on treatment (Table 1). A sputum cell count was available for 82/83 participants to assign inflammatory phenotype. Thirty-seven participants had eosinophilic asthma, defined as sputum eosinophils ≥3 % and 45 had non-eosinophilic asthma. Most participants with non-eosinophilic asthma (*n* = 28) had a paucigranulocytic phenotype (34 %); 17 had neutrophilic phenotype (21 %) and five participants had a mixed eosinophilic and neutrophilic phenotype (6 %).

**Table 1:** Patient characteristics

Number	83
Age, mean (range)	61 (21–82)
Sex, male (%)	42 (51)
BMI, mean (SD)	30.0 (5.5)
Atopy, n (%)	61 (73)
Ex-smoker, n (%)	34 (41)
Pack years, median (q1,q3)	26 (2,40)
FEV_1_ % predicted, mean (SD)	72.2 (20.3)
FEV_1_/FVC, mean (SD)	0.66 (0.12)
GINA treatment step n (%)	
3	14 (17)
4	67 (81)
5	2 (2)
ACQ6, mean (SD)	1.83 (0.82)
ICS dose μg, median (q1,q3)	1000 (800,2000)
Taking regular OCS, n (%)	3 (4)
Sputum eosinophils %, median (q1,q3)	2.1 (0.5,9.5)
Blood eosinophils, x10^9^/mL, median (q1,q3), *n* = 67	0.23 (0.11, 0.40)

*BMI* body mass index, *ACQ6* Asthma Control Questionnaire 6, *ICS* inhaled corticosteroid dose (in CFC beclomethasone equivalents), *OCS* oral corticosteroids

### Periostin levels in serum and sputum

For all subjects, periostin levels were significantly higher in serum (51.6 (41.8, 62.6) ng/mL) than in sputum (1.11 (0.5, 2.0) ng/mL) (*p* < 0.001).

Sputum periostin was assessed at three dilutions, 1 in 2, 1 in 10 and 1 in 50. A matrix effect (a direct or indirect alternation or interference in the measurement of an analyte) was observed on assay linearity with increased concentrations of periostin measured when the sample was more diluted (four representative results are shown in Table 2). To control for this effect, sputum periostin levels were analysed at a single dilution. Using the 1:2 dilution, sputum periostin was detectable in 65/83 (78 %) samples, where most samples (*n* = 59) had sputum periostin concentrations within the assay dynamic quantitative range. Six samples had periostin levels that were beyond the maximum of quantitative range at 1:2 and 17 samples had levels that were below the detection level. Alternately, using the 1:10 dilution, sputum periostin was detectable in 35/83 (42 %) samples; this is significantly fewer when compared with the proportion of samples detected using the 1:2 dilution (78 % vs. 42 % *p* = 0.045). Subsequent analysis of sputum periostin was undertaken using the results from the 1:2 dilution.

**Table 2:** Sputum periostin levels increased with increasing dilution of sputum supernatant

	Dilution		
ID	1:2	1:10	1:50
24312	0.35	0.49	0.95
24327	ND	0.17	ND
24488	0.27	0.49	1.78
24586	0.47	0.36	1.21

Periostin results are shown as ng of periostin per mL of sputum supernatant

*ND* not detected

We observed a reduction in periostin concentration assessed when periostin protein was diluted in a sputolysin containing diluent (see Additional file 1 and Additional file 2).

### Periostin levels and sputum eosinophils

**Fig. 1:**
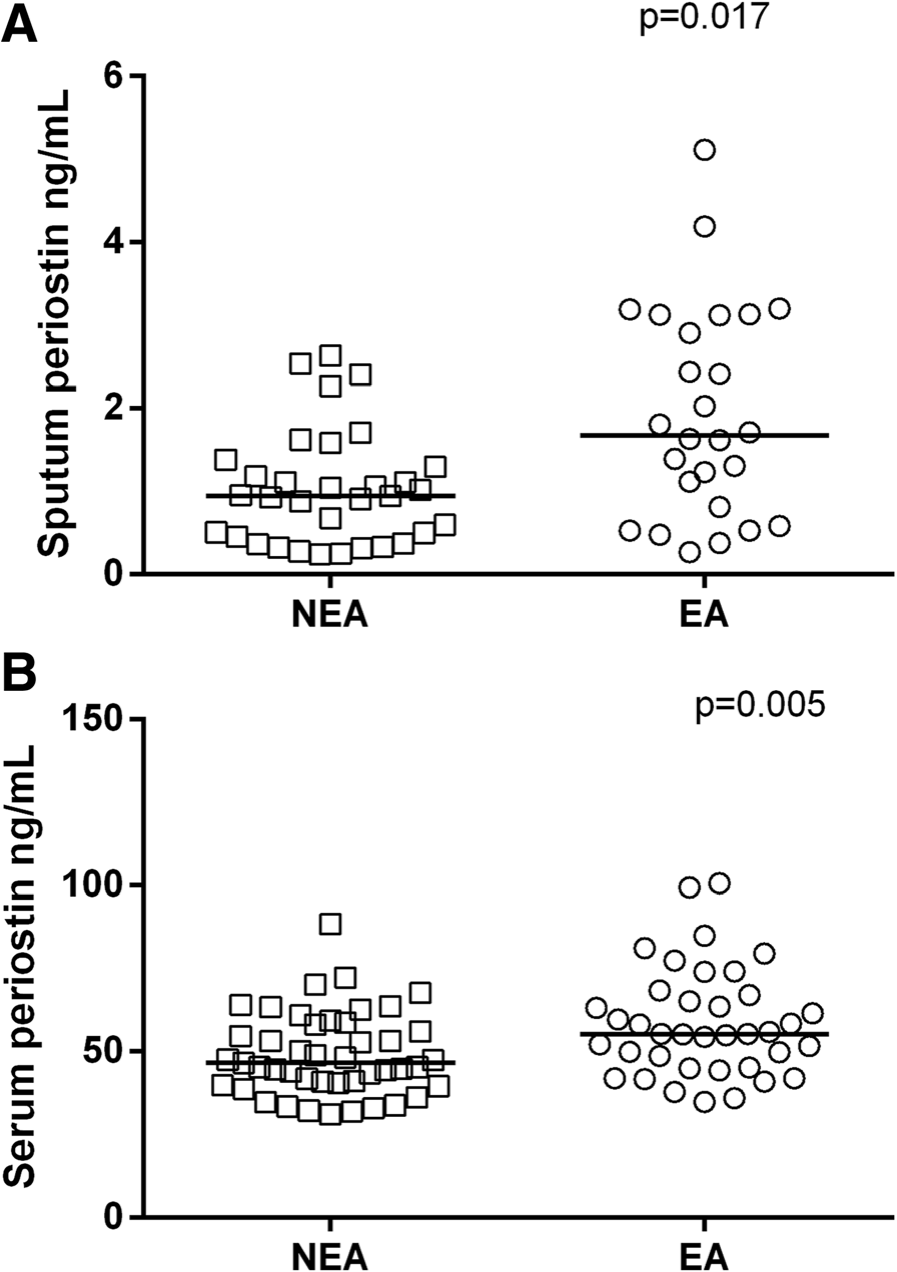
Periostin levels in participants with non-eosinophilic asthma (NEA, open squares) and eosinophilic asthma (EA, open circles) assessed in 55 sputum samples (**a**) and 77 serum samples (**b**). The data was analysed using STATA 11 software. The horizontal line represents the median value for each group. A rank-sum test was performed with significance indicated when *p* < 0.05

Sputum and serum periostin were significantly higher in patients with eosinophilic asthma compared with non-eosinophilic asthma (Fig. 1).

**Fig. 2:**
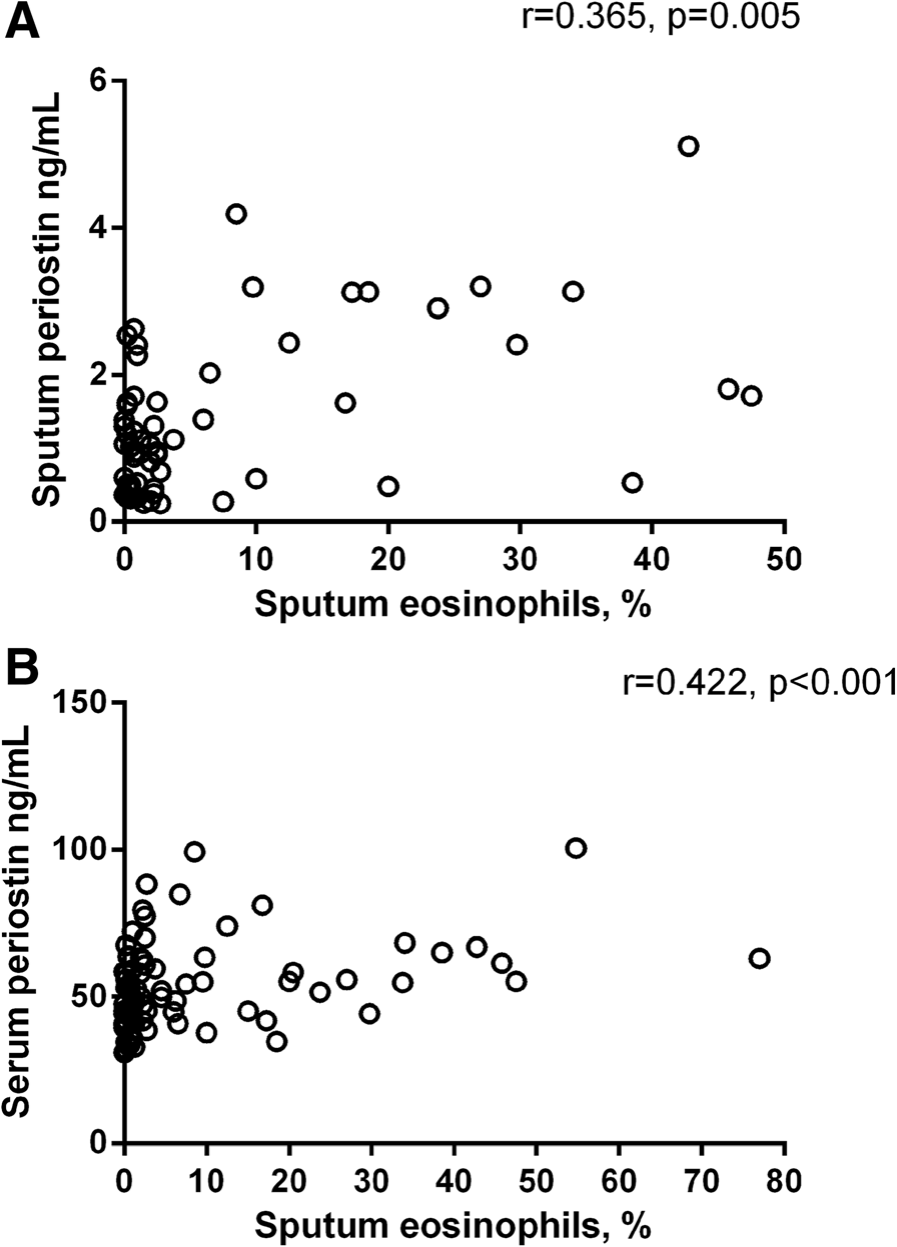
Scatter plots showing the association between sputum (**a**) and serum (**b**) periostin levels with sputum eosinophils. The data was analysed using STATA 11 software. Spearman’s rank correlation was used to assess the associations between sputum and serum periostin levels. Results were reported as significant when *p* < 0.05

Both serum and sputum periostin levels were significantly associated with sputum eosinophils (*r* = 0.422, *p* = 0.0001 and *r* = 0.364, *p* = 0.005 respectively, Fig. 2).

**Fig. 3:**
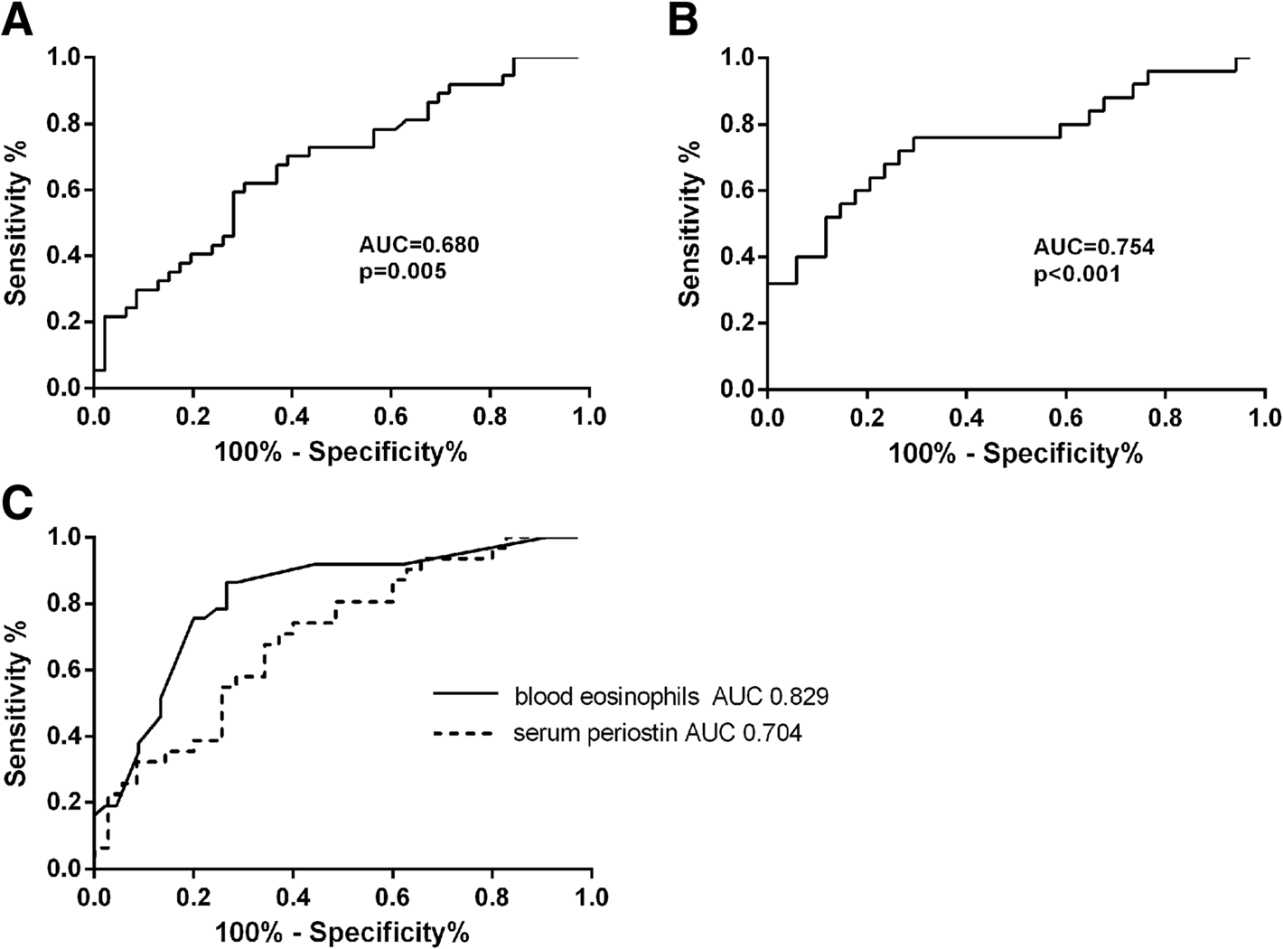
Receiver operating characteristic (ROC) curves. The area under the curve (AUC) for (**a**) serum periostin in 83 samples (**b**) sputum periostin 83 samples and (**c**) the comparison of serum periostin and blood eosinophils in the same 67 samples. The data was analysed using STATA 11 software. The performance characteristics of the periostin (**a** and **b**) and blood eosinophil (**c**) variables were examined by calculating AUC which was considered statistically significant when *p* < 0.05

In order to examine the utility of serum and sputum periostin to predict the presence of eosinophilic asthma (≥3 % sputum eosinophils) we generated ROC curves. The area under the curve (AUC) for sputum periostin was 0.754, *p* < 0.001, whereas for serum periostin the AUC was 0.680, *p* = 0.005 (Fig. 3). The AUC did not improve by using a 2 % sputum eosinophil cut-off point to distinguish eosinophilic from non-eosinophilic asthma (data not shown).

Peripheral blood eosinophil counts were available in only 67 participants. We compared the ability of blood eosinophils and serum periostin as a biomarker to predict the presence of eosinophilic asthma in the same 67 samples. Peripheral blood eosinophils had a more favourable AUC of 0.820 compared with an AUC of 0.704 for serum periostin, *p* = 0.086.

### Associations with clinical and inflammatory cell profile

There was no association between mean asthma control score (ACQ6) and either sputum or serum periostin levels (*r* = 0.038 and *r* = −0.062, p > 0.05 respectively). Similarly, there was no difference in ACQ6 in those with high serum periostin (≥50 ng/mL) or low serum periostin (<50 ng/mL) levels (median ACQ6 1.8 vs 1.7, *p* = 0.530). Both sputum and serum periostin were significantly and inversely associated with body mass index (BMI) (*r* = −0.368, *p* = 0.004 and *r* = −0.325, *p* = 0.003 respectively) but not associated with ICS dose, smoking pack years, lung function or atopy. There was no difference in sputum or serum periostin levels in never smokers compared with ex-smokers (data not shown). Using logistic regression there was no improvement in the model to explain the presence of eosinophilic asthma by the addition of BMI to serum periostin levels.

### BMI and periostin

Given the negative association observed between periostin levels and BMI we sought to determine if BMI impacted on the ability of serum periostin to predict the presence of eosinophilic asthma. The ability of serum periostin to predict the presence of eosinophilic asthma was not improved following correction for BMI, with a corrected AUC of 0.681, which was not statistically different to the uncorrected AUC of 0.680, *p* = 0.551.

## Discussion

In this well-characterised cohort with poorly-controlled asthma, periostin was detected in 78 % of sputum samples at a 1:2 dilution. While levels of periostin in induced sputum were lower than those measured in serum, both sputum and serum periostin levels were significantly associated with airway eosinophilia. Additionally, both sputum and serum periostin levels were significantly higher in patients with eosinophilic asthma compared with non-eosinophilic asthma. Our data are supported by similar correlations between serum periostin and sputum eosinophils in other cohorts [4, 14].

It is unclear if the lower sputum periostin levels compared to serum are the result of limitations in detecting proteins in sputum supernatant samples processed with sputolysin, differences in the assays utilised in the detection of periostin or a biological difference between the sample types. The ELISA that was used for the assessment of sputum periostin was validated for the measurement of serum periostin, but not sputum and the matrix effect observed could influence the lower levels reported. Also, while both the serum and sputum assays use the same antibody pair, they use different platforms and reagents, which may lead to differences in sensitivity. Serum periostin was measured previously by Genentech using both assay platforms in 195 severe asthma serum samples and on average Elecsys® Periostin values were 2.03 times (+/- 0.01 s.e.) greater than those derived by ELISA (unpublished data)*.* Since the assays have not been standardized against each other, the levels can not directly be compared. However, assay differences alone are unlikely to explain the 50-fold difference between sputum and serum periostin levels observed in this study and further investigation is required including an assessment of the variation of periostin levels over time in sputum.

There may also be a biological explanation for the observed lower levels of periostin in sputum. It has been shown that periostin is strongly expressed at the RNA level in epithelial cells but protein expression could not be detected in these cells [6, 7]. However, periostin is immunolocalized to the basement membrane immediately below the epithelial cells as well as mesenchymal cells [7]. This discrepancy between RNA and protein expression has led to the postulation that periostin protein is rapidly secreted by airway epithelial cells into the subepithelial layer. *In vitro* tissue culture experiments using an air/liquid interface, representing the airway lumen and bronchial tissue respectively, support this hypothesis, as periostin was abundantly present in the basal medium, but could not be detected in the apical washes [7]. It is therefore feasible that periostin may not be present in high concentrations in the sputum supernatant collected from the airway lumen. Alternately, the low sputum periostin levels may represent plasma leakage or the presence of periostin in the sputum supernatant may indicate a sub-group of patients who have a specific alteration in the epithelial barrier in some way or suggest a different cellular source of periostin in the sputum samples.

The utility of identifying eosinophilic asthma is well established with improved management and reduced exacerbations as key outcomes [1, 15]. However, sputum induction is not widely available and is unlikely to be adopted into routine clinical practice. As such, there is an unmet need for surrogate biomarkers to assist clinical decision making in asthma. Despite a statistically significant correlation between both serum and sputum periostin and the sputum eosinophil proportions, the strength of the association was relatively modest such that neither sputum nor serum periostin levels could sufficiently predict the presence of an eosinophilic inflammatory subtype with AUC in the poor to fair range [16]. We then compared blood eosinophils and serum periostin levels as surrogates to identify patients with eosinophilic asthma as identified by sputum eosinophils ≥3 %. In agreement with Wagener et al [17], we found blood eosinophils were an accurate predictor of patients with eosinophilic asthma (AUC 0.829) and serum periostin did not adequately distinguish eosinophilic from non-eosinophilic asthma in adults. Sputum periostin similarly lacked the rigor to be an adequate biomarker to detect eosinophilic asthma in adults with poorly-controlled asthma being neither sensitive nor specific and has the added disadvantage of not being easily accessible in clinical practice. In the BOBCAT study, serum periostin measured using the ELISA platform was found to be a sensitive but not specific marker of airway eosinophilia and performed better than blood eosinophils at predicting the presence of airway eosinophilia as defined by eosinophils in both sputum and tissue [4]. Of course, all studies so far including the current study reported here may be limited by the numbers of participants included with varying patient characteristics, levels of treatment and severity of asthma, and larger studies may provide more insight into the ability of serum periostin to predict the presence of eosinophilic asthma.

There are limited studies supporting the utility of periostin in the management of asthma. There is evidence that periostin levels can indicate better treatment outcomes in studies involving the IL-13 monoclonal antibody lebrikizumab or anti-IgE antobody omalizumab [8, 13, 18]. In these studies blood eosinophils and fractional exhaled nitric oxide (FeNO) were also able to identify people with greater benefit and future clinical studies need to determine how each of these biomarkers will be used in clinical practice. While periostin levels may not be suitable as surrogate markers for detecting the presence of eosinophilic asthma, its biomarker utility may lay in its ability to predict treatment response to anti-IL-13 therapy [13, 19], although this has yet to be tested in prospective studies.

## Conclusions

In poorly-controlled asthma treated with inhaled corticosteroids, periostin levels are low in airway secretions, but can be detected. Although sputum and serum periostin levels are significantly related to sputum eosinophil proportions, their ability to predict eosinophilic asthma in poorly-controlled asthma appears relatively modest.

### Consent for publication

Informed consent was obtained from the participants at the time of recruitment for publication of patient data where their identity was remained confidential.

### Availability of data and materials

The detailed method describing validation of periostin assay has been included in Additional file 2.

The dataset(s) supporting the conclusions of this article are shown as individual data points in Figs. 1, 2 and 3. An additional file with supplementary table has been provided that includes de-identified individual periostin and inflammatory phenotype data.

## Additional files

Additional file 1: Table S1. Inflammatory phenotype (sputum), serum and sputum periostin levels of individual patients included in the study. (DOCX 18 kb) Additional file 2: Periostin assay validation. (DOCX 13 kb)

## Abbreviation

ACQ6: Asthma Control Questionnaire 6
AUC: area under curve
BAL: bronchoalveolar lavage
BMI: body mass index
DTT: dithiothreitol
FeNO: fractional exhaled nitric oxide
FEV1: forced expiratory volume in 1 s
ICS: inhaled corticosteroids
IQR: interquartile range
ppm: parts per million
Th2: T-helper 2 cells
