# Supplementary material for: Periostin levels and eosinophilic inflammation in poorly-controlled asthma
Source: BMC Pulm Med. 2016 Apr 30;16:67. doi: 10.1186/s12890-016-0230-4 (PMC4851782; doi:10.1186/s12890-016-0230-4)
Supplement: Additional file 1: Table S1. — Inflammatory phenotype (sputum), serum and sputum periostin levels of individual patients included in the study. (DOCX 18 kb) [file 12890_2016_230_MOESM1_ESM.docx]

**Additional file 1: Table S1:** Inflammatory phenotype (sputum), serum and sputum periostin levels of individual patients included in the study.

| SNo. | ID | Inflammatory phenotype | Serum periostin (ng/ml) | Sputum periostin (ng/ml) |
| --- | --- | --- | --- | --- |
| 1 | 19900 | 1 | 35.82 | 1232.27 |
| 2 | 20512 | 0 | 43.76 | 1706.98 |
| 3 | 20586 | 0 | 58.08 | 1182.25 |
| 4 | 21032 | 1 | 55.15 | 475.88 |
| 5 | 21158 | 0 | 52.66 | 249.43 |
| 6 | 21560 | 0 | 62.55 |  |
| 7 | 21691 | 1 | 55.16 | 1710.24 |
| 8 | 21987 | 0 | 70.11 | 950.17 |
| 9 | 22273 | 0 | 50.08 | 1041.86 |
| 10 | 22282 | 1 | 61.44 | 1806.14 |
| 11 | 22415 | 1 | 55.12 |  |
| 12 | 22429 | 1 | 63.06 |  |
| 13 | 22505 | 0 | 48.07 | 1111.01 |
| 14 | 22552 | 0 | 47.40 |  |
| 15 | 22640 | 0 | 32.12 | 361.86 |
| 16 | 23374 | 0 | 39.39 | 1059.29 |
| 17 | 23378 | 0 | 58.52 |  |
| 18 | 23380 | 1 | 59.59 | 1118.99 |
| 19 | 23382 | 1 | 58.36 |  |
| 20 | 23447 | 1 | 58.03 | 811.22 |
| 21 | 23537 | 1 | 66.92 | 5118.35 |
| 22 | 23723 | 1 | 63.41 | 3194.05 |
| 23 | 23736 | 0 | 55.96 | 2538.51 |
| 24 | 24062 | 1 | 49.82 |  |
| 25 | 24086 | 0 | 38.58 | 676.44 |
| 26 | 24244 | 0 | 40.42 | 1381.70 |
| 27 | 24312 | 1 | 34.72 | 3128.49 |
| 28 | 24327 | 0 | 33.34 |  |
| 29 | 24488 | 1 | 44.17 | 2411.10 |
| 30 | 24586 | 1 | 99.36 | 4194.25 |
| 31 | 24732 | 0 | 39.71 | 599.58 |
| 32 | 24733 | 0 | 45.17 | 1298.96 |
| 33 | 24761 | 0 | 40.90 | 1110.14 |
| 34 | 24768 | 0 | 60.79 | 905.89 |
| 35 | 24770 | 1 | 51.60 | 2907.59 |
| 36 | 24784 | 1 | 65.12 | 527.08 |
| 37 | 24797 | 0 | 49.66 | 527.26 |
| 38 | 24861 | 1 | 55.76 | 3203.46 |
| 39 | 24875 | 1 | 52.01 |  |
| 40 | 24877 | 1 | 44.85 | 1394.79 |
| 41 | 24879 | 1 | 37.65 | 582.34 |
| 42 | 24882 | 0 | 44.17 | 370.76 |
| 43 | 24883 | 1 | 41.58 | 375.73 |
| 44 | 24886 | 0 | 41.71 | 1623.40 |
| 45 | 25029 | 0 | 44.45 | 877.43 |
| 46 | 25041 | 1 | 41.78 | 3124.91 |
| 47 | 25175 | 0 | 34.51 | 505.71 |
| 48 | 25220 | 0 | 33.67 |  |
| 49 | 25369 | 1 | 68.31 | 3134.54 |
| 50 | 25844 | 0 | 31.84 |  |
| 51 | 25855 | 0 | 43.34 | 2267.00 |
| 52 | 26258 | 0 | 53.12 | 1018.36 |
| 53 | 26270 | 0 | 54.52 | 2631.37 |
| 54 | 26335 | 1 | 100.60 |  |
| 55 | 26465 | 0 | 59.17 |  |
| 56 | 26504 | 0 | 63.53 | 313.36 |
| 57 | 26521 | 1 | 81.07 | 1617.89 |
| 58 | 26523 | 0 | 63.37 | 277.22 |
| 59 | 26526 | 0 | 32.86 | 926.45 |
| 60 | 27092 | 0 | 45.13 | 241.24 |
| 61 | 27232 | 0 | 67.58 | 323.14 |
| 62 | 27245 | 1 | 41.75 |  |
| 63 | 27500 | 0 | 63.88 | 498.29 |
| 64 | 30277 | 1 | 84.76 |  |
| 65 | 30450 |  | 74.11 |  |
| 66 | 30680 | 0 | 53.00 | 1580.38 |
| 67 | 30880 | 0 | 72.23 | 328.30 |
| 68 | 30940 | 0 | 46.44 | 2408.05 |
| 69 | 31190 | 1 | 77.30 | 1632.78 |
| 70 | 31690 | 1 | 47.25 | 452.57 |
| 71 | 31840 | 1 | 54.35 | 268.75 |
| 72 | 31885 | 0 | 44.68 | 941.38 |
| 73 | 31990 | 1 | 73.95 | 2436.01 |
| 74 | 32165 | 1 | 79.45 | 1309.30 |
| 75 | 32254 | 1 | 40.81 | 2028.95 |
| 76 | 33054 | 0 | 40.78 |  |
| 77 | 35554 | 1 | 54.86 |  |
| 78 | 36154 | 0 | 48.76 |  |
| 79 | 36404 | 0 | 30.88 |  |
| 80 | 37250 | 0 | 88.28 |  |
| 81 | 37850 | 1 | 45.09 |  |
| 82 | 38156 | 1 | 48.55 |  |
| 83 | 38277 | 0 | 36.03 |  |
