# Supplementary material for: Periostin levels and eosinophilic inflammation in poorly-controlled asthma
Source: BMC Pulm Med. 2016 Apr 30;16:67. doi: 10.1186/s12890-016-0230-4 (PMC4851782; doi:10.1186/s12890-016-0230-4)
Supplement: Additional file 2: — Periostin assay validation. (DOCX 13 kb) [file 12890_2016_230_MOESM2_ESM.docx]

**Additional file 2**

*Periostin assay validation*

The effect of DTT in sputum samples on the assay performance was assessed with simulated samples in which known amounts of periostin were added during processing. Briefly, different known levels of recombinant periostin were spiked into assay diluent, which contained different amounts of bovine serum albumin (BSA), mimicking the total protein amounts that can be present in sputum samples. The samples were treated with DTT and processed as for sputum processing. Samples were subsequently stored at room temperature or -80ºC for varying lengths of time. The amounts of periostin in the sputum samples were measured with the ELISA assay and the recovery calculated to estimate the effect of DTT. The results showed that effect of DTT on the assay performance was positively correlated with the sample incubation time and negatively correlated with the amount of total protein present in the sample. However, when up to 10mg/mL BSA was added to the assay diluent very limited effect of DTT on the quantitation of periostin was observed.
